# Internalization of negative societal views on old age into self-perceptions of aging: exploring factors associated with self-directed ageism

**DOI:** 10.3389/fsoc.2023.1291325

**Published:** 2023-11-17

**Authors:** Motoko Ishikawa

**Affiliations:** ^1^Faculty of Social Sciences, University of Helsinki, Helsinki, Finland; ^2^Department of Social Sciences and Philosophy, University of Jyväskylä, Jyväskylä, Finland

**Keywords:** ageism, self-directed ageism, negative age stereotypes, internalization, self-perceptions of aging

## Abstract

**Introduction:**

A growing number of research has provided evidence for the negative impact of ageism on older people’s health and well-being. Among the three different manifestations of ageism, namely institutional, interpersonal and self-directed ageism, significant ageism-health associations have been proved to be strongest for self-directed ageism. This supports stereotype embodiment theory, which maintains that lifetime exposure to negative age stereotypes leads to the internalization of ageism as a form of negative attitudes towards own aging and it adversely affects health and well-being in old age. However, little is known about how people internalize negative age stereotypes held in the society into self-perceptions of aging.

**Methods:**

This study aimed to explore how socially shared beliefs about old age are internalized into self-perceptions of aging focusing on uncovering factors related to self-directed ageism. Data were derived from the survey that had examined citizen’s attitudes towards old age and aging in Finland. Multinominal logistic regression models were performed to examine the association of sociodemographic and contextual factors with different combinations of societal age stereotypes and two indicators of self-perceptions of aging: subjective views on old age and personal feelings of own old age.

**Results:**

The analyses showed that being female, attaining tertiary education, evaluating poor quality of life and awareness of institutional old age discrimination were related to holding negative views on aging towards both society and oneself.

**Discussion:**

The findings from univariate and multivariate models suggest that it is not age *per se*, but structural and cultural circumstances shaped with growing older that turns socially shared negative age stereotypes into negative self-perceptions of aging. Even though the study addressed situations in one country, the findings have an important implication for other rapidly aging societies regarding how social and cultural contexts are closely linked to the formation of self-directed ageism.

## 1 Introduction

Ageism is a multifaceted social phenomenon that is defined as the stereotypes, prejudice and discrimination directed towards others or oneself based on age (World Health Organization, 2021). The three dimensions of ageism signifies that stereotypes affect how we think, prejudice affects how we feel, and discrimination affects how we act towards people on the basis of their age (Officer et al., 2020). Ageism can operate either explicitly (consciously) or implicitly (unconsciously), and it manifests itself at three different levels as institutional, interpersonal and self-directed ageism, being intertwined and mutually reinforcing (Marques et al., 2020; World Health Organization, 2021). Ageism is regarded to be prevalent, deeply ingrained and more socially accepted than other forms of bias, and older people are most vulnerable to ageism though it can affect any age group (Ayalon et al., 2019; Officer et al., 2020; World Health Organization, 2021).

Over the past two decades, a growing body of research has provided evidence for the negative impact of ageism on older people’s health and well-being. A first global systematic review on the impacts of ageism on health, which included both structural- and individual-level studies from 45 countries, found that ageism led to significantly worse health outcomes: 95.5% of the 422 studies and 74.0% of the 1,159 associations between ageism and health showed evidence of the adverse effects of ageism (Chang et al., 2020). Significant ageism-health associations were observed across 11 health domains: exclusion from health research, devalued lives of older persons, lack-of-work opportunities, denied access to healthcare and treatments, reduced longevity, poor quality-of-life and well-being, risky health behaviors, poor social relationships, physical illness, mental illness and cognitive impairment. These domains represent a broad range of health outcomes, which conforms with World Health Organization’s (2020) definition of health as a state of complete physical, mental and social well-being and not merely the absence of disease or infirmity. The systematic review also found that the association between ageism and health outcomes was strongest for self-directed ageism, that is, when ageism was turned against oneself and operationalized as self-perceptions of aging measure. For instance, negative self-perceptions of aging significantly reduced longevity (Zhao et al., 2017), increased risk of harmful tobacco and alcohol use (Villiers-Tuthill et al., 2016), and impeded recovery from severe disability (Levy et al., 2012). Moreover, a recent study revealed that the connection between COVID-19 health worries and anxiety symptoms was stronger among older adults with high levels of self-directed ageism (Bergman et al., 2020).

Other systematic review and meta-analysis of longitudinal studies corroborate the findings of adverse health effects of ageism among older adults. A systematic review, which examined the longitudinal consequences of self-perceptions of aging in participants 50 years or older by synthesizing 21 studies across five western countries, revealed that having more positive self-perceptions of aging was consistently associated with better self-rated health and less obesity, greater longevity, better performance of the activity of daily living, less depression and better cognitive functioning (Tully-Wilson et al., 2021). Self-perceptions of aging (SPA) refer to a person’s general evaluation of own aging process and reflect internalized stereotypes of aging. SPA and its reliable scale used in numerous longitudinal studies―the Attitude Towards Own Aging (ATOA) scale (Lawton, 1975; Liang and Bollen, 1983; Tully-Wilson et al., 2021) ―are regarded as a measure to appraise self-directed ageism. Thus, the results of this systematic review suggest that maintaining positive self-perceptions of aging provides a buffer against the negative impact of ageism on various health outcomes of older people. Likewise, a meta-analysis of 19 longitudinal studies found a small significant overall effect of subjective aging―operationalized either as subjective age or self-perceptions of aging―on health, health behaviors and survival over time (Westerhof et al., 2014). The analyses, however, revealed heterogeneity, with stronger effects on the more proximal outcome of health compared to survival, over a shorter time period, in younger participants in their second half of life, and in welfare regimes with less state provisions. Taken together, previous research including the recent systematic reviews as well as meta-analysis highlights that ageism is a global health issue, which constitutes an important, and hitherto neglected, social determinant of health (Officer et al., 2020; World Health Organization, 2021).

### 1.1 Theory on ageism-health associations

The ample evidence of significant ageism-health associations provides support for stereotype embodiment theory (SET). Drawing on research showing that positive and negative age stereotypes held by older individuals can have beneficial and detrimental effects, respectively, on a variety of cognitive and physical outcomes, SET proposes that age stereotypes are embodied when their assimilation from the surrounding culture leads to self-definitions that, in turn, influence functioning and health (Levy, 2009). There are four components constituting the theory. First, age stereotypes (i.e., beliefs about older people in general) typically begin to develop in childhood and are reinforced and internalized across the life span. Second, these stereotypes can operate unconsciously. Third, as one grows older, age stereotypes become increasingly relevant for the aging person when identifying oneself with old person. Fourth and the finally, these age stereotypes gained salience from self-relevance shape self-stereotypes of aging (Levy, 2003; Wurm et al., 2017) and they exert influence on an array of health outcomes through multiple pathways: psychologically, behaviorally, and physiologically. Accordingly, SET offers a lifespan perspective on the internalization and impact of both positive and negative age stereotypes on individual aging process (Wurm et al., 2017; Tully-Wilson et al., 2021), which occurs in a top-down way from society to the individual as well as over time from childhood to old age (Levy, 2009).

Significant associations between ageism and health as well as longitudinal health consequences of self-perceptions of aging identified in aforementioned studies specifically validate the third and fourth components of SET, namely the adverse effect of internalized negative age stereotypes or self-directed ageism on health and well-being in old age. In contrast, much fewer studies have focused on the ways in which age stereotypes, as socially shared beliefs about older people and old age, are incorporated into self-stereotypes. A comprehensive systematic review found a total of 14 determinants of ageism against older people, of which 13 associated robustly with other-directed forms of ageism, and only one had an effect on self-directed ageism (Ayalon and Tesch-Römer, 2017; Marques et al., 2020). Self-directed ageism was mostly determined by older adults’ mental and physical health status. This finding implies bi-directional nature of the association between self-directed ageism and health. Meanwhile, another study found lower healthy life expectancy and fewer proportion of older people within a country as explanatory factors for an individual or a country being ageist (Officer et al., 2020). Nevertheless, it is again relevant to ageism on interpersonal level but not to self-directed ageism.

To sum it up, little is known about processes between age stereotypes and self-stereotypes, especially in terms of how negative age stereotypes generally held in the society develop self-directed ageism via self-relevance. To date, no robust determinants of self-directed ageism have been found at the interpersonal/intergroup and institutional/cultural level of analysis (Marques et al., 2020). The scarce knowledge about determinants of self-directed ageism presents a major research gap, while the theory maintains that individuals assimilate age stereotypes from the surrounding culture (Levy, 2009), and specifically research has increasingly demonstrated detrimental effect of self-directed ageism on health and well-being. A sociological approach that sheds light on socio-cultural influences on individuals in shaping self-directed ageism is now called for.

### 1.2 The current study

To address the identified knowledge gap, the current study aims to examine under what conditions, including sociodemographic characteristics and socio-cultural contexts, people are susceptible for internalizing socially perceived beliefs about the process of aging and about older people. In other words, it investigates how socially shared beliefs about old age are linked to individuals’ self-perceptions of aging. The research questions are as follows: (1) Does increasing age cause socially shared negative age stereotypes to shape negative self-perceptions of aging among older adults? (2) If older age is not the major predictor of self-directed ageism, what other factors are associated with internalization of negative societal age stereotypes into self-perceptions of aging?

This study utilizes dataset from the survey in which participants with a broad age range were asked about their perceptions of age stereotypes shared in the society and self-perceptions of aging. Inquiring views on aging on both societal and individual levels has an advantage in undertaking the research questions of the present study. Besides, two questions introduced in the survey to assess self-perceptions of aging capture multifaceted nature of ageism: one is about respondents’ negative attitudes towards aging and the other refers to fear of aging. According to SET, lifetime exposure to negative age stereotypes leads to the internalization of ageism as a form of negative attitudes towards own aging. Meanwhile, terror management theory maintains that threats of death, physical deterioration, and insignificance evoked by the encounter with older adults manifest in ageism as a fear of aging directed towards others as well as selves (Martens et al., 2005; Lev et al., 2018). Thus, the two questions are both relevant to the manifestation of ageism, but it presumably emerges through different pathways. This suggests that these questions should be analyzed separately, since determinants of self-directed ageism may differ between the two.

Answering to the research questions by analyzing this survey data fills a gap that has been identified in both the theory (SET) and empirical studies. Given the accumulated knowledge on adverse health effects of ageism, the most notable is that results of this study about factors associated with self-directed ageism will highlight potential vulnerable population groups and life situations to which social- and health policy as well as interventions should pay more careful attention.

## 2 Materials and methods

### 2.1 Data

The survey examining citizens’ attitudes towards old age, aging and older population in Finland was the data source for this study. VTKL – The Finnish Association for the Welfare of Older Adults commissioned the survey to Aula Research Oy. The targets of the survey were Finnish citizens aged 16 years and older and sampling was conducted to correspond to the distribution of target population according to residential region, age, and gender. The survey was conducted between January and March 2022 through online questionnaire for all age groups and by telephone interviews for those who were 85+ years old (Vaarama, 2022). The response rate of the online survey was 19%, whereas 46% of people contacted by telephone responded to the questions.

The survey received 2,056 respondents. However, we removed 30 cases without information on gender or birth year and 29 cases who did not respond to the question on societal views on old age, 10 cases who did not answer the question on subjective views on old age, and 3 cases without response to the question on personal feelings of own old age. Additionally, we removed one case whose birth year was 1900 and 2 cases whose birth year was 2007 or later. Thus, we had 1,981 respondents in our final study cohort.

Given that recent rise in population aging seems to be associated with negative attitudes towards aging and older people in a global context (North and Fiske, 2015), Finland is an interesting country for investigating how age stereotypes shared in the society and people’s attitudes towards own aging are related. In Finland, as a country belonging to the Nordic welfare regime, public authorities have been responsible for universal provisions of income security and care services for older people. However, the country has been aging most rapidly in Europe (Eurostat, 2023a), which poses a serious challenge to upkeep the public provisions. Hence, the recent demographic change and its possible negative consequences in old age social policies may influence both societal and self-perceptions of aging.

### 2.2 Outcome variables

To examine the research questions on how socially shared beliefs about old age are linked to individuals’ self-perceptions of aging, this study created new outcome variables by merging values of two questions. The followings explain the original variables on societal age stereotypes and self-perceptions of aging and how these were integrated into the new variables.

#### 2.2.1 Age stereotypes shared in the society

Age stereotypes were assessed in the survey as people’s perceptions of societal views on old age by asking participants as follows: ‘How positively or negatively do you see old age is regarded in Finnish society in general?’ As the present study focuses on investigating negative perceptions, response alternatives with a six-level Likert scale were dichotomized as *negatively* (‘very negatively’ and ‘somewhat negatively’) and *positively or neutrally* (‘very positively’, ‘somewhat positively’, ‘neither positively nor negatively’ and ‘cannot say’).

#### 2.2.2 Self-perceptions of aging

Self-perceptions of aging were measured in the survey using two questions: one asked participants’ attitudes towards aging in a relatively general manner and the other explored more explicitly personal feelings of own old age. The first question, ‘How positively or negatively do you see old age?’ was intended for assessing subjective views on old age and this was inquired before the previously mentioned question on societal views on old age. The same six response alternatives as societal age stereotypes (from ‘very positively’ to ‘very negatively’) were recoded similarly in a dichotomous manner. The second question focused more on participants’ personal feelings regarding fear of aging: ‘When you think of your own old age, what does it feel like?’ To examine negative perceptions, five-level Likert scale for response was dichotomized as *feel fear* (‘very fearful’ and ‘somewhat fearful’) and *feel safe or neutral* (‘very safe’, ‘somewhat safe’ and ‘neither safe nor fearful’).

#### 2.2.3 Creating two outcome variables: combinations of societal age stereotypes and self-perceptions of aging

Next, the two outcome variables were created for the present study by merging previously described dichotomous variables. The variable on societal age stereotypes was merged with each variable on self-perceptions of aging: one with subjective views on aging (attitudes towards aging) and another with personal feelings of own old age (fear of own old age). The combined new variables had four categories: (1) those who take a positive/neutral stance towards both societal views on old age and self-perceptions of aging, (2) those who have negative societal views on old age, while self-perceptions of aging are positive/neutral, (3) those who have positive/neutral societal views on old age, while self-perceptions of aging are negative and (4) those who take a negative stance towards both societal views on old age and self-perceptions of aging. Respondents located in the last category refer to those who internalize negative age stereotypes in the society into self-perceptions.

### 2.3 Explanatory variables

Explanatory variables included the following demographic and socioeconomic characteristics: gender, age in ten-year groups, living arrangements (‘living with a partner’ or ‘not living with a partner’), highest attainment in education (‘basic education’, ‘secondary education’ or ‘tertiary education’) and income level (‘enough money for daily needs’ or ‘not enough money or reluctance to answer’). The cumulative advantage –disadvantage perspective has theoretically and empirically explained the interindividual divergence in a given characteristic (e.g., money, health, or status) with the passage of time, which reproduces a greater heterogeneity and inequality in late life (Dannefer, 2003). Thus, these explanatory variables were expected to be related to self-directed ageism shaped over the course of one’s life. Besides, gender is especially important in studies on ageism, as the so-called ‘double standard of aging’, referring to discrimination against older women both because of their gender and their age, has generated different meanings of aging for women and men (Sontag, 1978; Arber and Ginn, 1995).

Self-rated quality of life (‘very good’, ‘good’, ‘neither good nor bad’, ‘bad’ or ‘very bad’) was also included as an explanatory variable. Given the socio-cultural influence on shaping age stereotypes (Levy, 2009), participants’ opinions about whether following institutions are discriminating against older people were incorporated in the analysis: politicians, media, health care services (e.g., doctor’s appointment), social services (e.g., home care, service housing, meal service), the Social Insurance Institution of Finland, private companies (e.g., shops, pharmacies, restaurants), transport and employers.

### 2.4 Analysis

Multinomial logistic regression models were performed to examine the association of participants’ sociodemographic backgrounds, quality of life and opinions on old age discrimination with the two outcome variables (Kleinbaum and Klein, 2002). First, separate logistic models were fitted for each explanatory variable to analyze their unadjusted associations with the outcome variables (Model 1). Since all explanatory variables were categorical with k classes, they were transformed as k-1 dummy variables. For example, for education the equation is defined as:


(1) $$\mathrm{logit}\left(\pi \left(x\right)\right)=\beta_{0}+\beta_{1}x_{\mathrm{secondary}}+\beta_{2}x_{\mathrm{tertiary}}$$

where logit(π(x)) is the logarithmic transformation of the expected value of binary outcome variable, β_0_ is the intercept of linear predictor and β_1_ and β_2_ are the regression coefficients for the secondary and tertiary education dummy variables, respectively. The regression coefficients were transformed as relative risks (RR) thus describing the probability of having the value 1 of outcome variable for secondary and tertiary education as compared to basic education used as the reference category. Further, 95% confidence intervals (CI) for RRs were calculated using standard errors.

After that a full logistic regression model was fitted including all explanatory variables simultaneously (Model 2) to study how the mutual adjustment for other explanatory variables affects these associations. In the case of p explanatory variables, the equation is:


(2) $$\mathrm{logit}\left(\pi \left(x\right)\right)=\beta_{0}+\beta_{1}x_{1}+\beta_{2}x_{2}+\beta_{3}x_{3}+\ldots \beta_{p}x_{p}$$

Multinomial logistic regression model can be extended to have an outcome variable with k categories estimating k-1 sets of estimates. Since the outcome variables had 4 classes, three sets of regression estimates were estimated. In this case, RRs describe the probability to belong in a certain category of outcome variable as compared to the reference categories of both the outcome and explanatory variables. The models were estimated using Stata/SE 17.0 for Windows statistical software (StataCorp, College Station, TX, United States).

## 3 Results

### 3.1 Sample characteristics and share of having negative societal views on old age and having negative self-perceptions of aging

Table 1 displays characteristics of the total sample according to categorized groups and share of having negative societal views on old age and having negative self-perceptions of aging in respective groups of the sample. These are background information for the multinominal logistic regression models explained later. The share of those who had negative subjective views on old age and those who felt fear about own old age was smaller than the share of those who negatively perceived societal views on old age in almost all groups. This supports the argument that people endorse certain but not all socio-cultural age stereotypes (Wurm et al., 2017). It is also noteworthy that while older participants, except the oldest old, had more negative perceptions on how old age was regarded in the society, those who had negative subjective views on aging and were fearful about own old age tended to decrease as age of the respondents increased.

**Table 1:** Sample characteristics for the total sample and share of having negative societal views on old age and having negative self-perceptions of aging in respective groups.

	Distribution in the sample (%), *N* = 1,981	Share of having negative societal views on old age (%)	Share of having negative subjective views on old age (%)	Share of feeling fear about own old age (%)
*Gender*				
Male	47.7	36.7	14.2	22.1
Female	52.3	50.8	15.6	32.9
*Age in ten-year groups*				
16–25	11.3	30.0	16.1	30.0
26–35	12.4	37.4	17.5	37.8
36–45	13.2	37.0	15.3	36.6
46–55	14.8	42.7	13.7	33.8
56–65	18.9	51.5	18.1	29.1
66–75	17.0	54.2	11.6	18.2
76–85	8.9	54.8	11.3	10.2
86 +	3.5	29.0	14.5	10.1
*Living arrangements*				
Living with a partner	46.9	43.7	12.6	24.9
Not living with a partner	53.1	44.4	17.0	30.3
*Education*				
Basic education	19.9	37.2	13.7	26.6
Secondary education	54.6	43.3	14.7	28.9
Tertiary education	25.4	51.2	16.5	26.2
*Income level*				
Sufficient	48.1	40.4	12.9	18.4
Insufficient/ reluctance to answer	51.9	47.4	16.8	36.4
*Quality of life*				
Very good	8.7	28.9	5.8	6.9
Good	46.0	40.4	11.5	21.2
Neither good nor bad	32.2	46.9	15.7	31.4
Bad	11.1	59.4	30.1	53.4
Very bad	2.1	63.4	36.6	68.3
*Institutions involved in old age discrimination*				
Politicians				
No	71.4	34.6	13.6	23.0
Yes	28.6	67.7	18.2	39.8
Media				
No	86.9	40.2	14.8	26.1
Yes	13.1	69.9	16.2	38.6
Health care services				
No	76.7	37.4	13.6	24.9
Yes	23.3	66.2	19.5	37.1
Social services				
No	74.1	36.9	13.2	23.1
Yes	25.9	64.5	19.9	41.1
The Social Insurance Institution of Finland				
No	81.3	39.5	13.7	23.7
Yes	18.7	63.9	20.5	45.3
Private companies				
No	92.6	42.0	14.0	26.7
Yes	7.4	70.1	27.2	40.8
Transport				
No	90.3	41.4	14.3	26.0
Yes	9.7	69.3	21.4	44.3
Employers				
No	77.7	37.0	12.7	23.6
Yes	22.3	68.8	22.6	42.3

### 3.2 Association of different factors with combinations of societal age stereotypes and subjective views on old age

Table 2 presents the results of multinominal logistic regression analyses that investigated the association of explanatory variables with combinations of societal age stereotypes and subjective views on old age. Reference category was those who took a positive/neutral stance towards both societal views on old age and subjective views on old age. The results show the likelihood of being placed in other three categories (negative societal views–negative subjective views, negative societal views–positive/neutral subjective views, positive/neutral societal views–negative subjective views) in respect of respondents’ characteristics compared with the reference category. Model 1 refers to univariate analysis in which each variable was included into the model separately, whereas multivariate analysis controlled for all variables is shown in Model 2.

**Table 2:** Association of individual characteristics and contextual factors with combinations of societal age stereotypes and subjective views on old age.

Categories^a^	Negative societal views on old age–Negative subjective views on old age		Negative societal views on old age–Positive/neutral subjective views on old age		Positive/neutral societal views on old age–Negative subjective views on old age	
	Model 1^b^	Model 2^c^	Model 1	Model 2	Model 1	Model 2
Variables	RR^d^ (95% CI)	RR (95% CI)	RR (95% CI)	RR (95% CI)	RR (95% CI)	RR (95% CI)
*Gender*						
Male	ref.	ref.	ref.	ref.	ref.	ref.
Female	**1.62 (1.20–2.19)**	**1.89 (1.36–2.63)**	**1.83 (1.50–2.24)**	**2.08 (1.66–2.60)**	1.02 (0.65–1.59)	0.97 (0.61–1.55)
*Age in ten-year groups*						
16–25	ref.	ref.	ref.	ref.	ref.	ref.
26–35	1.61 (0.83–3.14)	0.92 (0.45–1.88)	1.28 (0.83–1.99)	0.85 (0.53–1.36)	0.84 (0.42–1.68)	0.79 (0.38–1.67)
36–45	1.46 (0.75–2.84)	0.73 (0.35–1.50)	1.25 (0.82–1.92)	0.78 (0.49–1.25)	0.63 (0.31–1.30)	0.54 (0.25–1.18)
46–55	1.57 (0.82–3.01)	0.76 (0.37–1.53)	**1.63 (1.08–2.46)**	1.06 (0.67–1.65)	0.48 (0.22–1.03)	**0.37 (0.16–0.84)**
56–65	**2.87 (1.58–5.21)**	1.49 (0.78–2.86)	**2.09 (1.41–3.10)**	1.38 (0.90–2.13)	**0.40 (0.18–0.87)**	**0.30 (0.13–0.70)**
66–75	**1.98 (1.05–3.74)**	1.31 (0.66–2.60)	**2.61 (1.76–3.88)**	**1.97 (1.28–3.04)**	**0.18 (0.06–0.54)**	**0.15 (0.05–0.47)**
76–85	1.79 (0.85–3.78)	1.32 (0.59–2.92)	**2.84 (1.81–4.45)**	**2.26 (1.39–3.68)**	0.36 (0.12–1.09)	0.32 (0.10–1.02)
86 +	1.13 (0.42–3.07)	0.98 (0.34–2.81)	0.83 (0.42–1.64)	0.82 (0.40–1.70)	0.60 (0.20–1.86)	0.45 (0.14–1.47)
*Living arrangements*						
Living with a partner	ref.	ref.	ref.	ref.	ref.	ref.
Not living with a partner	1.25 (0.93–1.69)	1.20 (0.86–1.68)	1.04 (0.85–1.26)	1.09 (0.87–1.36)	**2.10 (1.30–3.39)**	1.61 (0.97–2.68)
*Education*						
Basic education	ref.	ref.	ref.	ref.	ref.	ref.
Secondary education	1.41 (0.93–2.16)	1.59 (1.00–2.51)	1.22 (0.94–1.59)	1.28 (0.96–1.71)	0.81 (0.48–1.38)	0.96 (0.54–1.68)
Tertiary education	**2.04 (1.28–3.23)**	**2.98 (1.77–5.00)**	**1.65 (1.23–2.21)**	**1.99 (1.42–2.80)**	0.76 (0.39–1.47)	1.02 (0.49–2.10)
*Income level*						
Sufficient	ref.	ref.	ref.	ref.	ref.	ref.
Insufficient/ reluctance to answer	**1.65 (1.22–2.23)**	0.91 (0.62–1.33)	**1.26 (1.04–1.53)**	1.02 (0.79–1.31)	1.17 (0.75–1.82)	0.68 (0.40–1.16)
*Quality of life*						
Very good	ref.	ref.	ref.	ref.	ref.	ref.
Good	**2.83 (1.20–6.67)**	**3.16 (1.31–7.65)**	**1.56 (1.07–2.27)**	**1.52 (1.01–2.30)**	1.86 (0.65–5.37)	2.09 (0.72–6.10)
Neither good nor bad	**4.61 (1.95–10.88)**	**4.73 (1.90–11.77)**	**1.98 (1.35–2.91)**	**1.69 (1.09–2.64)**	2.69 (0.92–7.82)	**3.74 (1.23–11.43)**
Bad	**13.58 (5.55–33.26)**	**14.74 (5.59–38.85)**	**2.96 (1.85–4.74)**	**2.79 (1.63–4.79)**	**6.52 (2.10–20.26)**	**9.92 (2.97–33.12)**
Very bad	**19.83 (6.15–63.96)**	**21.14 (5.93–75.38)**	**3.69 (1.57–8.64)**	**3.22 (1.25–8.28)**	**10.82 (2.37–49.33)**	**16.26 (3.25–81.31)**
*Institutions involved in old age discrimination (ref.: not discriminating)*						
Politicians	**3.66 (2.66–5.04)**	**1.56 (1.02–2.38)**	**4.05 (3.24–5.08)**	**2.32 (1.73–3.12)**	1.01 (0.56–1.84)	1.12 (0.52–2.41)
Media	**2.80 (1.84–4.28)**	0.81 (0.48–1.37)	**3.54 (2.62–4.79)**	1.29 (0.89–1.86)	0.64 (0.23–1.80)	0.58 (0.18–1.80)
Health care services	**3.64 (2.62–5.06)**	1.52 (0.96–2.40)	**3.10 (2.45–3.94)**	1.39 (1.00–1.93)	0.81 (0.41–1.61)	0.86 (0.36–2.05)
Social services	**3.57 (2.59–4.92)**	**1.58 (1.01–2.47)**	**3.00 (2.39–3.78)**	1.37 (1.00–1.87)	1.12 (0.62–2.00)	1.36 (0.62–2.98)
The Social Insurance Institution of Finland	**3.32 (2.35–4.69)**	0.99 (0.61–1.60)	**2.49 (1.93–3.22)**	0.88 (0.62–1.25)	0.86 (0.42–1.77)	0.67 (0.27–1.66)
Private companies	**5.03 (3.12–8.12)**	**2.35 (1.31–4.23)**	**2.77 (1.85–4.16)**	1.22 (0.75–1.99)	1.23 (0.43–3.52)	1.11 (0.34–3.62)
Transport	**3.72 (2.38–5.82)**	0.94 (0.53–1.64)	**2.99 (2.12–4.24)**	1.03 (0.67–1.57)	0.88 (0.31–2.49)	0.77 (0.24–2.49)
Employers	**4.95 (3.55–6.91)**	**2.60 (1.73–3.92)**	**3.57 (2.79–4.58)**	**1.84 (1.36–2.49)**	1.45 (0.79–2.65)	1.69 (0.82–3.51)

Values showing the significance level of *p* < 0.05 are bolded.

a Those who have positive/neutral attitudes towards both societal views on old age and subjective views on old age as reference category (*n* = 1,024, 51.7%). Distribution of other categories: negative societal views on old age–negative subjective views on old age (*n* = 212, 10.7%), negative societal views on old age–positive/neutral subjective views on old age (*n* = 661, 33.4%), positive/neutral societal views on old age–negative subjective views on old age (*N* = 84, 4.2%).

b All variables included into the model separately.

c All variables included into the model simultaneously.

d Ratio of probability of outcome in respective categories vs reference category.

The primary focus of this study was to examine who had negative perceptions of societal views on old age and hold negative subjective views on old age as well. Multivariate analysis controlled for all variables revealed that respondents who were female, had the highest educational level, rated quality of life as worse, and saw politicians, social services, private companies and employers as perpetrators of ageism were more likely to think that old age was regarded negatively in Finland and were themselves liable to view aging in a negative light. It can be argued that people with such characteristics are susceptible for internalizing negative societal age stereotypes into their own perceptions about aging. However, as the table presents, other categories of the outcome variable also had many predictors with statistical significance. Hence, the following subsections describe findings to highlight the role of each factor in the associations.

#### 3.2.1 Gender

Being female increased the risk of considering both societal and subjective views on old age as negative, as well as perceiving negative societal attitudes towards aging while holding positive/neutral subjective views on old age. Multivariate analysis controlled for all variables even strengthened the gender effect for these categories. However, gender was not related to the likelihood of viewing old age negatively on a personal level despite having positive/neutral societal views on aging. The findings indicate that negative attitudes among females were directed mainly to societal beliefs about aging rather than to their own views on old age.

#### 3.2.2 Age

Respondents aged between 56 and 75 years compared with the youngest age group were more likely to perceive aging negatively on both societal and individual levels. Age-related changes in one’s life course such as retirement, approaching old age, and care needs for close relatives and/or oneself might make respondents think in this manner. However, adjusting for other variables attenuated association effect of age. Older age also increased the risk of having negative societal views on aging while subjectively viewing old age in a positive/neutral light. This applied to the respondents aged 46 years and older except the oldest old. Multivariate analysis controlled for all variables kept the significant association for age between 66 and 85. Conversely, both univariate and multivariate analyses revealed that older respondents were less likely to negatively view old age on a personal level despite holding positive/neutral societal views on old age. Age effects on all categories of the outcome variable suggest that older people were inclined to shape negative perceptions of aging towards society and much unlikely towards themselves.

#### 3.2.3 Living arrangements

Whether the respondents lived with a partner or not was generally not related to different combinations of societal age stereotypes and subjective views on old age.

#### 3.2.4 Education

Compared to those who had only basic education, respondents with tertiary education were more likely to perceive old age as negative on both societal and personal levels, as well as to have negative societal views on old age while subjective views were positive or neutral. Adjusting for other variables even increased the association effect of the highest education for these categories. However, educational level was not related to the category of having positive/neutral societal views on old age and simultaneously holding negative subjective views on old age. The results indicate that negative attitudes towards aging among people with tertiary education were directed mainly to the society rather than to themselves.

#### 3.2.5 Income level

When income level was analyzed separately from other variables, living under insufficient economic situation increased the risk of belonging to the two categories: negative societal views on old age–negative subjective views on old age, and negative societal views on old age–positive/neutral subjective views on old age. However, adjusting for other variables attenuated association effect of income level.

#### 3.2.6 Quality of life

Among different explanatory variables, poor quality of life appeared to be most robustly associated with respondents’ negative attitudes towards aging on both societal and individual levels. The multivariate analysis revealed that the risk of having such way of thinking was 15 times higher for those who evaluated quality of life as bad and 21 time higher for those with very bad quality of life, compared with people with very good life quality. Poorer self-rated quality of life was significantly related to the other two categories too, but the risk was higher for negatively perceiving subjective views on old age while having positive/neutral societal view on aging. This indicates that those who rated worse quality of life were more likely to shape negative perceptions of aging towards themselves rather than towards the society.

#### 3.2.7 Institutions involved in old age discrimination

The univariate analysis showed that seeing each social institution as discriminating against older people increased the risk of negatively perceiving aging on both societal and individual levels, as well as holding negative societal views on aging while subjective views were positive/neutral. Though adjusting for other variables attenuated most of the association effects, regarding politicians, social services, private companies and employers as perpetrators of ageism remained to be significantly associated with having negative perceptions of both societal and subjective views on old age. Meanwhile, opinions on old age discrimination were not related to the likelihood of viewing old age negatively on a personal level despite having positive/neutral societal views on aging. These findings suggest that whether each institution was discriminating against older people was more relevant with respondents’ perceptions of societal views on old age than their self-perceptions of aging.

### 3.3 Association of different factors with combinations of societal age stereotypes and personal feelings of own old age

Table 3 shows the association of respondents’ sociodemographic and contextual factors with combinations of societal age stereotypes and personal feelings of own old age. Reference category was those who took a positive/neutral stance towards societal views on old age and felt safe or neutral about their own old age. The likelihood of being placed in other three categories (negative societal views–feeling fear about own old age, negative societal views–feeling safe/neutral about own old age, positive/neutral societal views–feeling fear about own old age,) in comparison to the reference category was specified in the table.

**Table 3:** Association of individual characteristics and contextual factors with combinations of societal age stereotypes and personal feelings of own old age.

Categories^a^	Negative societal views on old age–Feeling fear about own old age		Negative societal views on old age–Feeling safe/neutral about own old age		Positive/neutral societal views on old age–Feeling fear about own old age	
	Model 1^b^	Model 2^c^	Model 1	Model 2	Model 1	Model 2
Variables	RR^d^ (95% CI)	RR (95% CI)	RR (95% CI)	RR (95% CI)	RR (95% CI)	RR (95% CI)
*Gender*						
Male	ref.	ref.	ref.	ref.	ref.	ref.
Female	**2.31 (1.79–2.98)**	**2.84 (2.11–3.82)**	**1.81 (1.46–2.25)**	**2.11 (1.66–2.69)**	**1.89 (1.39–2.58)**	**1.99 (1.42–2.80)**
*Age in ten-year groups*						
16–25	ref.	ref.	ref.	ref.	ref.	ref.
26–35	**2.48 (1.41–4.38)**	1.37 (0.73–2.58)	0.76 (0.46–1.26)	**0.51 (0.30–0.87)**	0.78 (0.47–1.29)	0.64 (0.37–1.12)
36–45	**2.37 (1.35–4.16)**	1.12 (0.60–2.11)	0.76 (0.46–1.25)	**0.48 (0.28–0.82)**	0.74 (0.45–1.22)	**0.50 (0.29–0.87)**
46–55	**2.50 (1.44–4.35)**	1.07 (0.58–2.00)	1.12 (0.71–1.78)	0.72 (0.44–1.19)	0.65 (0.39–1.08)	**0.38 (0.22–0.67)**
56–65	**2.80 (1.64–4.79)**	1.16 (0.63–2.13)	**1.66 (1.09–2.53)**	1.06 (0.67–1.69)	**0.38 (0.22–0.66)**	**0.21 (0.11–0.38)**
66–75	**1.86 (1.05–3.27)**	1.01 (0.54–1.91)	**2.18 (1.43–3.30)**	1.55 (0.98–2.44)	**0.17 (0.08–0.34)**	**0.11 (0.05–0.24)**
76–85	0.96 (0.46–2.02)	0.55 (0.25–1.22)	**2.61 (1.64–4.15)**	**1.92 (1.16–3.17)**	**0.13 (0.04–0.36)**	**0.08 (0.03–0.25)**
86 +	0.56 (0.20–1.57)	0.43 (0.14–1.35)	0.76 (0.39–1.50)	0.66 (0.32–1.36)	**0.10 (0.02–0.44)**	**0.07 (0.02–0.31)**
*Living arrangements*						
Living with a partner	ref.	ref.	ref.	ref.	ref.	ref.
Not living with a partner	1.24 (0.97–1.59)	1.04 (0.78–1.39)	1.03 (0.83–1.27)	1.10 (0.86–1.40)	**1.50 (1.10–2.05)**	1.04 (0.74–1.48)
*Education*						
Basic education	ref.	ref.	ref.	ref.	ref.	ref.
Secondary education	1.39 (0.99–1.95)	1.48 (1.00–2.20)	1.20 (0.90–1.61)	1.28 (0.93–1.76)	0.96 (0.66–1.40)	1.00 (0.66–1.52)
Tertiary education	**1.59 (1.09–2.34)**	**2.17 (1.37–3.43)**	**1.73 (1.26–2.39)**	**2.18 (1.51–3.14)**	0.74 (0.46–1.18)	0.95 (0.56–1.62)
*Income level*						
Sufficient	ref.	ref.	ref.	ref.	ref.	ref.
Insufficient/ reluctance to answer	**2.77 (2.13–3.60)**	1.30 (0.94–1.82)	1.08 (0.87–1.33)	0.89 (0.68–1.17)	**2.37 (1.72–3.26)**	1.03 (0.70–1.50)
*Quality of life*						
Very good	ref.	ref.	ref.	ref.	ref.	ref.
Good	**3.40 (1.68–6.90)**	**3.44 (1.63–7.25)**	**1.54 (1.05–2.27)**	**1.57 (1.03–2.40)**	**6.21 (1.92–20.06)**	**6.54 (2.00–21.41)**
Neither good nor bad	**6.26 (3.07–12.73)**	**5.41 (2.50–11.72)**	**1.99 (1.33–2.98)**	**1.90 (1.20–3.03)**	**12.00 (3.71–38.79)**	**14.47 (4.34–48.28)**
Bad	**20.65 (9.63–44.31)**	**20.08 (8.63–46.75)**	**2.93 (1.73–4.95)**	**3.17 (1.75–5.77)**	**29.80 (8.80–100.97)**	**40.94 (11.53–145.37)**
Very bad	**38.10 (12.74–113.91)**	**34.43 (10.28–115.26)**	2.51 (0.80–7.90)	2.62 (0.76–8.98)	**45.71 (9.90–211.16)**	**63.64 (12.83–315.69)**
*Institutions involved in old age discrimination (ref.: not discriminating)*						
Politicians	**5.86 (4.44–7.74)**	**2.33 (1.59–3.40)**	**3.72 (2.89–4.79)**	**2.23 (1.61–3.10)**	**1.81 (1.25–2.62)**	1.44 (0.87–2.38)
Media	**4.28 (3.03–6.07)**	1.33 (0.85–2.10)	**2.76 (1.97–3.86)**	0.92 (0.61–1.39)	0.81 (0.43–1.54)	0.58 (0.28–1.21)
Health care services	**4.14 (3.11–5.52)**	1.05 (0.70–1.60)	**3.05 (2.34–3.97)**	1.44 (1.00–2.06)	1.32 (0.87–2.00)	0.62 (0.35–1.10)
Social services	**4.98 (3.76–6.60)**	**1.78 (1.19–2.65)**	**2.93 (2.26–3.80)**	1.40 (0.98–1.98)	**2.10 (1.46–3.03)**	1.52 (0.90–2.57)
The Social Insurance Institution of Finland	**4.99 (3.67–6.79)**	1.31 (0.85–2.01)	**2.45 (1.81–3.31)**	0.99 (0.67–1.48)	**2.46 (1.65–3.67)**	**1.92 (1.10–3.35)**
Private companies	**4.23 (2.71–6.59)**	1.16 (0.65–2.06)	**2.62 (1.68–4.07)**	1.31 (0.77–2.23)	1.01 (0.46–2.21)	0.52 (0.21–1.27)
Transport	**4.94 (3.30–7.40)**	1.09 (0.65–1.84)	**2.80 (1.88–4.20)**	1.00 (0.62–1.63)	1.75 (0.96–3.17)	1.10 (0.54–2.26)
Employers	**5.99 (4.45–8.06)**	**2.66 (1.83–3.86)**	**3.47 (2.62–4.60)**	**1.97 (1.40–2.75)**	**1.98 (1.32–2.97)**	**1.73 (1.05–2.86)**

Values showing the significance level of *p* < 0.05 are bolded.

a Those who have positive/neutral attitudes towards societal views on old age and feel safe or neutral about their own old age as reference category (*n* = 908, 45.8%). Distribution of other categories: negative societal views on old age–feeling fear about own old age (*n* = 350, 17.7%), negative societal views on old age–feeling safe/neutral about own old age (*n* = 523, 26.4%), positive/neutral societal views on old age–feeling fear about own old age (*n* = 200, 10.1%).

b All variables included into the model separately.

c All variables included into the model simultaneously.

d Ratio of probability of outcome in respective categories vs reference category.

Multivariate analysis controlled for all variables revealed that respondents who were female, had the highest educational level, rated quality of life as worse, and considered politicians, social services and employers discriminating against older people were more likely to think that old age was regarded negatively in the society and were fearful about their own old age. These factors appeared to be associated with internalization of negative societal age stereotypes into self-perceptions of aging. However, since other categories of the outcome variable also had many significant predictors, findings are described according to the explanatory variables as follows.

#### 3.3.1 Gender

Being female increased the risk of being placed in all the three categories of the outcome variables. Multivariate analysis controlled for all variables even strengthened the gender effect for all the categories. The results suggest that compared to males, females were more likely to think that socially shared beliefs about aging were negative and they were also inclined to feel fear about own old age.

#### 3.3.2 Age

Respondents aged between 26 and 75 years compared with the youngest age group were more likely to perceive aging negatively on societal level as well as to feel fear about own old age. However, adjusting for other variables attenuated association effect of age. In terms of having negative societal views on old age while feeling safe/neutral about own old age, older age increased the risk of being placed in this category. In contrast, both univariate and multivariate analyses revealed that older respondents were obviously less likely to feel fear about own old age while having positive/neutral societal views on old age. The results of age effect indicate that older age was relevant to shaping negative perceptions of societal age stereotypes and was much unlikely to be related to fear of aging.

#### 3.3.3 Living arrangements

Whether the respondents lived with a partner or not was generally not related to different combinations of societal age stereotypes and personal feelings of own old age.

#### 3.3.4 Education

Attaining tertiary education was significantly associated with being placed in the two categories: negative societal views on old age–feeling fear about own old age, and negative societal views on old age–feeling safe/neutral about own old age. Adjusting for other variables even increased the effect of tertiary education. However, educational level was not related to the category of having positive/neutral societal views on old age and simultaneously feeling fear about own old age. This indicates that negative attitudes among people with tertiary education were directed mainly to societal beliefs about aging rather than to personal feelings of own old age.

#### 3.3.5 Income level

Univariate analysis revealed that living under insufficient economic situation increased the risk of belonging to the two categories: having negative societal views on old age–feeling fear about own old age, and having positive/neutral societal views on old age–feeling fear about own old age. This may indicate that disadvantage in economic situation was subject to fear of own old age rather than negative perceptions of societal views on aging. However, adjusting for other variables attenuated association effect of income level.

#### 3.3.6 Quality of life

Poor quality of life appeared to be the most decisive factor that made respondents hold negative societal views on old age and simultaneously feel fear about their own old age. In the multivariate analysis, the risk was 20 times higher for those who evaluated quality of life as bad and 34 times higher for those with very bad quality of life, compared with people with very good life quality. The effect of quality of life was even stronger on the category of having positive/neutral societal views on old age–feeling fear about own old age. This suggests that worse self-rated quality of life was more vulnerable to fear of own old age rather than to negative perceptions of aging on societal level.

#### 3.3.7 Institutions involved in old age discrimination

The univariate analysis revealed that seeing each social institution as perpetrators of ageism increased the risk of having negative societal views on old age and feeling fear about own old age, as well as having negative societal views on aging while feeling safe/neutral about own age. Considering politicians, social services, the Social Insurance Institution of Finland and employers as discriminating against old people was also significantly associated with being placed in the category of having positive/neutral societal views on old age–feeling fear about own old age. Though adjusting for other variables attenuated most of the association effects, involvement of old age discrimination by the institutions that seemed to be influential on societal level (politicians and employers) and on personal level (social services, the Social Insurance Institution of Finland and employers) remained to be significant.

## 4 Discussion

This study aimed to explore how socially shared beliefs about old age are internalized into self-perceptions of aging. It especially focused on uncovering factors related to self-directed ageism by investigating the association of sociodemographic and contextual factors with different combinations of societal age stereotypes and two indicators of self-perceptions of aging: subjective views on old age and personal feelings of own old age. While subjective views on old age referred to respondents’ own attitudes towards aging, personal feelings of own old age were used to estimate fear of own old age. Negative response, respectively, had to do with self-directed ageism.

The analyses showed that being female, attaining tertiary education, evaluating poor quality of life and awareness of institutional discrimination against older people were related to holding negative views on aging towards both society and oneself. Given the increasing evidence about adverse health effects of self-directed ageism, it can be argued that people with these attributes are inclined to internalize negative societal age stereotypes into self-perceptions, and hence are at risk of facing health and well-being related problems in old age.

The findings suggest that it is not age *per se*, but structural and cultural circumstances shaped with increasing age and through life course that turns socially shared negative age stereotypes into negative self-perceptions of aging. To put it another way referring to the theory of SET (Levy, 2009), self-relevance, which is considered to intervene in the internalization of age stereotypes into self-stereotypes, is not induced by age itself but by socio-cultural factors being intertwined with growing older. It means that different contexts surrounding aging constitute factors associated with self-directed ageism. This idea is relevant to understandings of age as socially and culturally constructed, being in interaction with other categorizations, such as gender, race and social class (Settersten and Hagestad, 2015; Ayalon and Tesch-Römer, 2018; Krekula et al., 2018). With these notions, ageism including self-directed ageism can be conceived as actively created in social encounters and processes (Krekula et al., 2018).

Significant associations of being female and having higher educational level with internalization of negative societal views on old age imply that these two variables may become risk factors for detrimental health effects from ageism. Focusing on educational attainment, this conflicts with previous research on social determinants of health that has demonstrated that better access to education positively influences health outcomes (Raghupathi and Raghupathi, 2020). Meanwhile, gender difference in consequences of health is not consistent: Although women generally live longer than men, they also live more years with functional disabilities and suffer more often from depressive disorders (Carmel, 2019). One possible explanation for the discrepancy between the present study and previous research is that women and people with higher education in Finland are most aware of increasing inequalities within the Nordic welfare regime caused by rapid demographic change, especially in terms of deteriorating quality of old age care policy and long-term care services (Hoppania et al., 2022; Rostgaard et al., 2022). Frequent encounter with ongoing public discussions on reform of healthcare, social welfare and rescue services (Finnish Government, 2023), the biggest nationwide care scandals happened in 2019 (Roslund and Mäntymaa, 2019), and serious understaffing in social and health sector seemed to negatively affect perceptions of societal views on old age and self-perceptions of aging in female and highly educated respondents.

Women’s negative attitudes towards both societal views on old age and self-perceptions of aging can also be explained by the concept of gendered ageism (Itzin, 1995; Krekula et al., 2018) that puts older women in a disadvantageous position in relation to career, pension, aging bodies and so on. Awareness of and presumably experience of gendered ageism among female respondents were specifically reflected in the higher risk of feeling fear about their own old age, compared to male respondents. Women were more fearful when they thought about personal aging, regardless of how positively or negatively they perceived societal age stereotypes.

Even though attaining higher education was associated with taking a negative stance towards both societal views on old age and self-perceptions of aging, negative attitudes were directed mainly to societal ageism. This is explained from the findings that people with tertiary education were more likely to negatively perceive societal views on old age independent of own self-perceptions of aging. Contrastingly, evaluating poor quality of life, which was also proved to be related to negative attitudes on both societal and individual levels, touched more upon personal understandings about aging, as respondents who rated worse quality of life were more likely to have negative attitudes towards own old age independent of their perceptions of societal ageism. These results suggest that higher education and poor self-rated quality of life had different implications for self-directed ageism. Namely, those who evaluated poor quality of life tended to individualize ageism, while people with higher educational level might be more resourceful to protect themselves from self-directed ageism, notwithstanding their critical attitudes towards societal views on aging.

The analysis revealed that worse self-rated quality of life was the most robust determinant of self-directed ageism. Quality of life is a useful concept to capture multifaceted dimensions of well-being. For instance, Eurostat has defined following dimensions as a framework for the measurement of quality of life: material living conditions, productive or main activity, health, education, leisure and social interactions, economic and physical safety, governance and basic rights, natural and living environment, and overall experience of life (Eurostat, 2023b). However, without specifying these multidimensional indicators, the concept tends to become obscure, which is the case for the survey used in the present study. Poor self-rated quality of life in this study could be understood as worry about and unsatisfaction with living conditions, considering that the survey did not include health related questions, occupational status was excluded from the analyses because it was not a suitable variable for respondents with a broad age range, and the income level was proved to be non-significant in the multivariate analysis. Future research should thoroughly investigate association between quality of life measured with a multidimensional scale and self-directed ageism.

This study included two indicators of self-perceptions of aging, namely subjective views on old age and personal feelings of own old age, in separate analyses. The reason for the separate analyses was that negative answers to these questions (=negative attitudes towards own aging, fear of own old age) signify the manifestation of multifaceted nature of ageism. Regarding internalization of negative societal views on old age into self-perceptions, significant factors were identified to be largely similar for both indicators. However, when examining the category of positive/neutral societal views on old age–negative self-perceptions of aging, being female, rating worse quality of life and awareness of institutional old age discrimination turned out to be more relevant to feeling fear about own old age, rather than to having negative subjective views on old age. This suggests that fear of aging may be the concept that more explicitly embodies the creation of ageism encountered by social and cultural factors (Ayalon and Tesch-Römer, 2018; Krekula et al., 2018).

Several limitations should be mentioned. First, due to the cross-sectional nature of the data, this study was not able to establish causality. Instead, only some significant associations were identified between participants’ characteristics and internalization of societal age stereotypes into self-perceptions. Accordingly, further investigation employing longitudinal designs is needed to validate the findings. Second, because of small sample sizes, the data did not have enough statistical power to conduct interaction analyses to test whether certain combinations of explanatory variables are associated with the outcome variables. Third, the measure used in this study to assess societal age stereotypes and self-perceptions of aging was different from scales that had been frequently used in previous research. The “Expectations Regarding Aging” (Sarkisian et al., 2005) as a measure for age stereotypes and the “Attitude Towards Own Aging (ATOA)” (Lawton, 1975; Liang and Bollen, 1983) for self-perceptions of aging have been demonstrated to be comprehensive scales for appraising ageism (Ayalon et al., 2019; Tully-Wilson et al., 2021), whereas outcome variables in this study were created by combinations of two simple questions. Hence, the findings from the analyses are not comparable to previous studies. Related to this, due to the nature of the survey to inquire attitudes towards old age and aging from citizens aged 16 years and older, the data were very general compared to those of research that exclusively investigate self-perceptions of aging. Finally, since the study addressed the situation in a single country, it is somewhat difficult to generalize the findings to other social contexts.

Nevertheless, this study addressed the knowledge gap on determinants of self-directed ageism and highlighted how social and cultural contexts were closely linked to internalization of negative societal age stereotypes into self-perceptions. Even though the discovered factors associated with self-directed ageism pertain to the Finnish society only, the findings have an important implication for other developed countries that are also facing aging of the population. In light of the understanding that rapid population aging tends to intensify negative societal age stereotypes, which is likely to cause aggravation of self-directed ageism and its adverse effects on health and well-being, it is crucial to specify people whose aging intersects with vulnerable or precarious conditions in structural, social and cultural terms. Concrete interventions to these people in social- and health policy will not only improve their well-being but also cater to alleviating social costs in the long run.

## Data availability statement

The data analyzed in this study is subject to the following licenses/restrictions: The data are not readily available as they can only be accessed by those who exchanged a written contract with the proprietor of the data. Requests to access these datasets should be directed to VTKL – The Finnish Association for the Welfare of Older Adults, https://vtkl.fi/in-english.

## Author contributions

MI: Conceptualization, Formal analysis, Methodology, Writing – original draft.
